# “It's My Country I'm Playing for”—A Biographical Study on National Identity Development of Youth Elite Football Players With Migrant Background

**DOI:** 10.3389/fspor.2022.893019

**Published:** 2022-07-12

**Authors:** Klaus Seiberth, Ansgar Thiel, Jannika M. John

**Affiliations:** ^1^Institute of Sports Science and Kinesiology, University Stuttgart, Stuttgart, Germany; ^2^Institute of Sports Science, University of Tübingen, Tübingen, Germany

**Keywords:** national identity development, elite sport, narrative, national team, youth elite football, migrant background, identity work, mapping

## Abstract

Sport represents a prominent topic for public and scientific debates on national identity. Looking at Germany, public discourses on national belonging have primarily focused on national elite football and on German-born international football players with Turkish background. Representing the biggest ethnic community in Germany and being highly represented in German youth elite football, athletes with Turkish background are prime examples for the complexity and ambiguity of identity formations in modern immigration countries in general and in youth elite football in particular. Current research has particularly focused on national identity formations of (youth) elite players with migrant background. However, there is a lack of studies that address the process of national identity development in youth elite sport. For that reason, the study aimed to explore stories of national identity development from the perspective of youth football players with Turkish background in German youth elite football. By conducting 10 expert interviews and biographical mappings, we identified specific types, strands, and trajectories of national identity development. Overall, we identified three types of narratives on national identity development: “going with the nomination(s),” “reconsidering national belonging,” and “adding up chances”. Our findings illustrate that national identity development in youth elite sport is particularly shaped by youth elite sport and the national team question. Hence, the stories indicate that international careers not necessarily foster national identification with a nation but can also reduce feelings of national belonging sustainably.

## Introduction

Within the last decades sport has become a prominent topic for public and scientific debates on national identity. In sport research, the topic of *national identity* has mostly been linked with aspects of (post-)migration, particularism and (re)nationalization (Hargreaves, 2002; Wong and Trumper, 2002; Bruce and Wheaton, 2009; Topič and Coakley, 2010; Maguire, 2011; Bairner, 2015). While sport is defined as “a hugely important marker of national identity” (Bairner, 2015, p. 378), football is supposed to be “a key area in expressing national belonging” (Dóczi, 2011, p. 2). Consequently, several studies aim to trace collective constructions of nationhood that are attached to or symbolized by national elite football (Maguire and Burrows, 2005; Wise, 2011; Griggs and Gibbons, 2014; Chiu, 2021). One of the main findings is that athletes are considered, at least in public perception, “representatives of the nation” and “iconic figures (…) in the international sporting arena” (McGee and Bairner, 2011, p. 439). Similarly, research on German elite football shows that the German male national football team serves as a symbol for collective constructions of national identity, for ethnic diversification, and for a “modern sense of national identity and a playful, non-threatening patriotism” (Merkel, 2014, p. 248).

Looking at Germany, public discourses on national belonging have primarily focused on German-born international players with Turkish background^1^ who—with regard to their international career—had the option to choose between playing for Germany as their country of birth and Turkey as their ancestors' country of origin. Knowing that the German Football Association (DFB) competes with the Turkish Football Federation (TFF) for German-born players with Turkish background on a junior level, it is obvious that these highly talented junior players are confronted with the question of which *nation* they want to play for within their international (junior) football career (Seiberth's et al., 2017; Seiberth and Thiel, 2021).

Accordingly, athletes with Turkish background are highly interesting cases for migration research. This is not just because they represent the biggest ethnic immigration community in Germany but also because the Turkish community has a very special status in the German public. This status is closely linked with Germany's immigration history and politics. When Turkish “guestworkers” started immigration to Germany in the course of the labor agreement in 1961, they were—in contrast to the guestworkers from Italy, Spain or Portugal—perceived as prime examples for cultural difference and religious otherness. In the 1970s, the public and media images of Turkish immigrants became increasingly negative. Since then, the Turkish community has often been labeled as a problem group in German public, media, politics, and science (Sökefeld, 2004; Thelen, 2016). While people with Turkish background are particularly confronted with disadvantages in some of the relevant fields of German society (Skrobanek, 2009; Kaas and Manger, 2012; Canan and Foroutan, 2016), they are, at the same time, highly represented in German (elite) football and in the various national junior squads of the German Football Association (DFB).

Interestingly, German-born international players with Turkish background seem to be under intense public scrutiny. Particularly, within the last years, there has been an ongoing debate about the question whether German internationals with Turkish background should be obliged to sing the national anthem in order to demonstrate their sense of belonging to and identification with Germany^2^. Besides, public debates have also focused on players with Turkish background who decided not to play for the DFB but for the TFF. These debates often assume a lack of national identification with Germany as the country of birth. Hence, media debates frequently revolve around the national team question and whether the choice of German-born players with Turkish background to play for Germany indeed symbolizes German national identity (Seiberth et al., 2018; van Campenhout and van Houtum, 2021).

Against this background, players with a Turkish background represent a typical *integration paradox*. On the one hand, the players become subjects of public debates on patriotism, notions of Germanness and national identification. On the other hand, national football teams of the DFB have become symbols for the ethnic diversification of German society and the chances that go along with transnational migration processes (Blecking, 2008; Meier and Leinwather, 2013; Merkel, 2014; Kaelberer, 2017). It is this paradox that makes German-born players with Turkish background an excellent subject of research for examining processes of national identity development in German immigration society.

Within academic research, athletes with migrant background and with bi-national references represent interesting case examples for interrelations between (post-)migration, national identity and elite sport. For example, Grainger's study on New Zealand's rugby team (Grainger, 2006), McGee and Bairner's (2011) study on (Northern) Irish football and Seiberth's et al. (2017) work on German youth elite football point to a fluidity of national identity indicating that practices of otherness, public notions of nationhood and athletes' experiences during their international career can affect athletes' constructions of national identity. On the one hand, this fluidity is closely related to what is called “sporting pragmatism“ (McGee and Bairner, 2011, p. 442). Being nominated by a federal sport organization can have an impact on personal constructions of national identity just as having negative personal experiences with a national football association. On the other hand, the studies illustrate that experiences of othering and experiences of not feeling fully accepted as a member of a national group or a national team have the potential to irritate athletes' national affiliations. According to this line of research, (elite) sport can be considered as “a significant contributor to the formation and sustenance of national and other identities” (Bradley, 2006, p.1202).

While current research has significantly contributed to understanding national identity formations in the context of (youth) elite sport, most studies exclusively focus on individual's current state of national identity. Consequently, there is a lack of studies that adopt a processual perspective particularly addressing the process of national identity development in (German) youth elite sport from the athlete's perspective. This is even more surprising as junior athletes with migrant background represent prime examples for the complexity and ambiguity of “identity work” (Andersson, 2002) in modern immigration countries.

Assuming that national identity formations in elite sport are the provisional result of a process that implies developmental trajectories and takes place under specific conditions in elite sport, our article focuses on national identity *development* in German junior elite football. Intending to contribute to elite sport and migration research we ask how national identity develops in German-born youth international football players with Turkish background and how the national team question affects player's national identity development. For this purpose, 10 stories on national identity development of German-born youth international football players with Turkish background have been analyzed to reconstruct national identity development within the players' (international) career by analyzing players' narratives and mapped memories. Based on a reconstructive approach we used narrative analysis to identify specific types, strands and trajectories of national identity development in German youth elite football.

## Theoretical Background

To identify types and strands of national identity development in German youth elite sport, we chose an *identity theoretical approach*. Although, identity theories have come up with various definitions, there is a broad consensus that identity is a diverse, dynamic and idiosyncratic construction of the self. Evidently, this construction is closely linked to the question of “how we perceive and define ourselves” (Turner, 2010, p. 16). In line with this conceptualization, we understand identity “as a subjective, constructed, and evolving story of how one came to be the person one currently is” (McLean and Syed, 2015, p. 320). In this sense, identities result from subjectively processing experiences, social contexts (such as youth elite sport) and cultural conditions. This process of exploring, reflecting and negotiating is assumed to be the condition for identity development. Although, childhood and adolescence are crucial developmental periods, identity is conceptualized as a life-span process. Generally, identity theories assume that this process is not fixed in an essentialistic way, but rather fragile, mutable, transitory, and constantly in progress (Schimank, 1988; Giddens, 1991; Hogg et al., 2004; Turner, 2010).

Therewith, identities are “signifiers of the self” (Ezzell, 2009, p. 111) that report on the current status of identity development. When intending to reconstruct this process of identity development (and not just single formations of identity), it is therefore necessary to understand the individual story. For that reason, biographic approaches rely on stories as a source for reconstructing developmental processes. In current narrative approaches “personal narratives” are relational constructions by which a person positions him- or herself to what is called “master narratives” (McLean and Syed, 2015). While master narratives represent “culturally shared stories that tell us about a given culture, and provide guidance for how to be a ‘good' member of a culture”, personal narratives in the form of stories “negotiate with and internalize these master narratives” (McLean and Syed, 2015, p. 320).

In this sense, identity is an “interactional accomplishment” (Cerulo, 1997, p. 387) that emerges from a “dialogical process through which we negotiate the implicit and/or explicit identity ascriptions we encounter in everyday life” (Andersson, 2002, p. 85). This process of “identity work” is supposed to be multidimensional as individuals permanently “work” on different components of the self. For that reason, (social) identity theories assume that “[p]eople have as many social identities and personal identities as there are groups that they feel they belong to or personal relationships they have” (Hogg et al., 2004, p. 252). *Social identity* is defined as “that part of the individuals' self-concept which derives from their knowledge of their membership of a social group (or groups) together with the value and emotional significance of that membership” (Tajfel, 1981, p. 255). One typical features of social identity is context dependency. Thus, “social identity is context dependent not only in terms of which social identity is salient but also in terms of what form the identity may take” (Hogg et al., 2004, p. 252). The concept of salience explains how different social identities relate to each other. It is assumed, that in any social context and in any situation, there is one specific social identity that takes the lead (Hogg et al., 2004). In this respect, however, it is noted that social identity “seems to be ‘switched on' by certain situations in ways that we do not as yet fully understand” (Turner, 2010, p. 21).

Reconstructing national identity development means to focus on one specific component of social identity resulting from a sense of belonging to a nation. In this context, the nation state is supposed to be “one of the most important agents of identification” (Brubaker and Cooper, 2000, p. 16). According to current research, *national identity* is based on a “shared sense of nation-hood grounded in the images and stories associated with an identifiable nation-state or longstanding ethnic population” (Topič and Coakley, 2010, p. 373/374). Typically, national identity comprises cognitive and emotional aspects of identification (Brubaker and Cooper, 2000; David and Bar-Tal, 2009). Obviously, constructions of national identities are associated with “particularistic configurations of ethnic cores, myths and memories, religious beliefs, language, connections with territory, and political values” (Moran, 2011, p. 2155).

National identity development starts in (late) childhood and consolidates in adolescence. For youths and adolescents with migrant background, national identity development turns out to be a complex developmental task. This is because it is closely linked to the exploration of a person's ethnic origin and his or her affiliations with the ancestors' country of origin and the own country of birth. Consequently, national identity results from subjectively exploring these relations and positioning oneself toward national groups (Phinney, 1992; Berry et al., 2006; Barrett, 2013). This process of exploration is assumed to be highly individual, dynamic and not predictable, which can also be seen within German migration research. Immigrants (of Turkish origin) and immigrant descendants with Turkish background in particular, often develop hybrid identities, which can imply strong feelings of belonging to both countries at the same time. These mixed and highly individualized identity formations represent typical phenomena of modern immigration societies—such as Germany (Faas, 2009; Wippermann and Flaig, 2009; Foroutan, 2013).

Knowing that national identity is context dependent and being “switched on” by certain situations, it must be considered that (youth) elite sport represents a social context providing specific conditions and constellations for (national) identity development. One reason for this is, that youth elite sport requires an extreme form of inclusion. This “hyperinclusion” (Göbel and Schmidt, 1998, p. 111) results from an extraordinary level of obligations linked to elite sport. Being socialized into elite sport, young athletes tend to integrate elite sports contextual logic prominently into their self-concept. This is particularly evident in German junior elite football. As competition is very high due to a large number of young talented players, players are expected to invest huge personal and time resources into football to increase the likelihood to succeed. The increasing internalization of master narratives of elite sport (such as having to subordinate all aspects of their lives to sports) lead (young) athletes into an “identity tunnel” (Curry, 1993, p. 289). Having entered this “tunnel”, *athlete identity* often becomes the dominant self-concept of (young) athletes with a potentially sustainable impact on sport-related decisions such as the national team question (Schubring and Thiel, 2014; Seiberth's et al., 2017).

A second reason why (youth) elite sport provides specific conditions for social identity development is its attachment to the concept of nation. Elite sport in general and youth elite football in particular is organized in national units and promoted by national football associations that compete with each other on the level of national teams. For example, being included into German youth elite football means to participate in the Talent Development Program of the German Football Association (DFB). Typically, these players train in and play for German Youth Performance Centers (YPC)—usually on the highest national level of competition. Playing for a German club usually also means that players are recruited for DFB state teams before they become international players. Generally, national talent promotion is always aiming to produce international players who represent their nation, nation-state or national football association. At the same time, becoming an international player is one of the highest achievements in (youth) elite sport. For players with migrant background this constellation not just includes a major challenge on the level of decision making but can also initiate a process of reflecting and “reworking” one's national identity. Generally, it can be assumed: “Through the life course, people extend, personalize, and revise their national identities as they connect themselves to stories told across multiple institutional spheres, including family, religion, politics, education, science, economics, and sport” (Topič and Coakley, 2010, p. 374).

While personal constructions and narratives of national identity are typically not public but rather private, they become an object of public interest when athletes with migrant background have to choose between two nations, which oftentimes becomes part of public and media debates. These public and media debates might provide master narratives that are received and processed by young athletes with a migrant background. Typically, within these debates the issue of national identity is linked to moral assessments such as “player eligibility” (Hassan et al., 2009). Therewith, the national team question turns out to function as a public visualizer of national identity. At the same time, it points to the relevance of media debates for the process of “encouraging the ‘imagined communities' of nations” (Ward, 2009, p. 527).

In German media debates, the choice of a national team has been instrumentalized as a question of emotional identification with the country of birth claiming national identity to be a major predictor for the players' choice. Furthermore, the public debates on German-born internationals with Turkish background often imply a low emotional identification with German society and “a virulent Turkish nationalism which finds its level of identification precisely in football” (Blecking, 2008, p. 965)^3^. We assume that players with migrant background take note of these debates and potentially integrate them in their self-concept.

We conclude that *national identity development* in elite sport takes place under very specific conditions in which sport specific narratives, public and media narratives, and broader sociocultural narratives must be reconciled. Under these conditions, ‘identity work' in general and ‘national identity work' in particular become very challenging for adolescent athletes with Turkish background. However, the national team question, the experiences during national training camps or international games, conversations with national coaches or representants of the national football associations can also represent special occasions to productively deal with personal and public constructions of national identification.

In order to reconstruct individual processes of national identity development in German junior elite football, research relies on stories in which changes, transitions, and switches on the level of national identification are linked with biographical experiences, career events, and contextual conditions. Life and career events, situations, and personal experiences are all assembled within the story plot that can provide insights into the storytellers' perspectives on the subject. The individual stories can also reflect broader narratives on national identity and the national team question (cf. Smith and Sparkes, 2009; McLean and Syed, 2015).

## Methods and Materials

The study is based on a *narrative research approach* combining guided expert interviews and biographical mappings comprising 10 case studies with male German-born international football players with Turkish background aged 15–21. Overall, our research is informed by an interpretative paradigm highlighting the multiple and subjective nature of reality (Poucher et al., 2020). In this regard, we aimed to assess the subjective meanings that young football players with a Turkish background attribute to their experiences of having to choose for which national team they want to play and how this decision has impacted their national identity development.

### Participants

We focused on players with Turkish background for two reasons: Firstly, people with Turkish background represent the largest immigrant population in Germany; secondly, the public and media debate on the national team question in Germany almost exclusively refers to players with Turkish background—such as Mesut Özil (Seiberth's et al., 2017)—and thereby might provide powerful master narratives surrounding the national team question and national identity for German-born players with Turkish background.

In order to find potential interviewees, we conducted a web search using the online database www.transfermarkt.de. This website provides a wide range of relevant data on elite (junior) football players in German (junior) teams. By concentrating on the feature nationality, we identified numerous top-level players who were characterized as being both “German” and “Turkish”. Case selection was based on the following criteria: The players were expected to have a Turkish background, to have been born in Germany (but not necessarily have German citizenship)^4^, to be between 15 and 21 years old, and train at a Youth Performance Center (YPC) of the DFB. A further essential requirement was that the players had played at least one international match during their career—either for the Turkish Football Federation or the German Football Association. In order to avoid a systematic distortion of results due to sampling bias, we aimed to select players from three different status groups: players who had played only for the German Football Association (DFB), players who had only played for the Turkish Football Federation (TFF), and players who had played for the German Football Association (DFB) and the Turkish Football Federation (TFF) during their youth career—which was in accordance with FIFA's eligibility regulations^5^ (FIFA, 2016).

We identified 31 players who matched the selection criteria. In a next step we officially contacted the YPCs in their function as gatekeepers. We informed them about the aim of the study, the specific target group, and the players we aimed to interview. Subsequently, the YPCs examined our request and autonomously decided whether to inform the respective players and their parents. If the YPCs assessed our request positively, we received either date proposals for the interviews or the contact details of the players to arrange an interview on-site. In this way, we respected the competences of the YPCs. At the same time, we avoided ethical concerns by directly influencing the players in their decision to participate in the study.

During the recruitment process, we experienced typical problems researchers face when attempting to recruit (youth) elite athletes for research purposes (Bairner, 2015). These problems were primarily associated with the players' hyperinclusion into youth elite sport. Having to coordinate youth elite football with a school career makes youth elite athletes a group with very limited time resources. However, we were not just dependent on the athletes' commitment to participate in the study but also on the willingness of the gatekeepers (YPC) to support the study. Although the recruitment and scheduling process was difficult and time-consuming, several YPCs and 10 top-level players responded positively to our request. Comparable sample sizes have also been used in prior published narrative analysis studies (Busanich et al., 2016; Cavallerio et al., 2017; Everard et al., 2021). As narrative studies aim to embrace the complexities and ambiguities inherent to experiences (Papathomas and Lavallee, 2012), small sample sizes are quite common.

### Data Collection

The overall aim of the study was to reconstruct the decision-making process of young football players who had to choose whether they want to play for a German or a Turkish national team on a junior level. Within the context of the overall study, we aimed to identify relevant reasons for this decision and asked for the role of ethnic and national identity in this decision-making process. As we assumed that national identity changes during the youth elite football career, the study also aimed to reconstruct trajectories of national identity development and to locate this process over the course of the players' football career. The present article focuses on the latter objective and presents multi-methodological data about this topic.

To reconstruct national identity development over the player's career, we employed a *multi-method design* that generated verbal data and graphical data by combining guided expert interviews with biographical mappings. We chose guided expert interviews as this method opens “windows into peoples' lives” (Denzin, 1989, p. 14) and provides detailed insights into experiences, relevant career events, and courses of development. Biographical mappings were used to support the athletes in their biographical “memory work” (Schubring et al., 2019, p. 1). Biographical mappings consist of a two-dimensional grid, with the x-axis representing a timeline and the y-axis representing an intensity scale ranging from 0 to 10. We used the biographical mapping technique for the reconstructive visualization of the sports careers, the national team decision-making process, and the development of national identity over the player's career. Being embedded in the interviews, the tool allowed to visualize life and career experiences on a timeline and “to express gradual differences in subjective experience” (Schubring et al., 2019, p. 1). The aim of this participatory visual method was to identify “trajectories, biographical turning points, and intersections of development strands” (Schubring et al., 2019, p. 1).

To keep players' time expenditure to a minimum and to use familiar surroundings, the interviews and the mappings were conducted at the players' club facilities. Since the players were born and socialized in Germany and spoke German fluently, the interviews were carried out in German. Data collection was initiated with an open question asking the interviewees to state important career events and experiences. This also included events and experiences, which referred to the national team question and the players' international career. In a next step, the interviewees were asked to locate these experiences on the timeline (x-axis) and to plot a *sporting success* line on the y-axis. The career events and biographic experiences were also the frame for separately mapping the players' *identification with Germany* and their *identification with Turkey* within their career using the intensity scale on the y-axis. Finally, interviewees explained the line drawings by particularly focusing on changes and turning points. The combination of interviews and mappings “allows interviewees to narrate and ‘map' strands of development within their life courses” (Schubring et al., 2019, p. 1). The interviews and mappings lasted between 45 and 90 min.

The interviews and mappings were prepared, organized and conducted by the lead author who does not have a (Turkish) migrant background. Although it has to be assumed that “pure objectivism is a naïve quest” (Bourke, 2014, p. 3), we constantly reflected on aspects of subjectivity and positionality. This was even more important as the interviews included several sensitive topics (such as family immigration history, ethnic identity or national identification). In order to encourage the players to express their views freely and in order to avoid socially desirable answers, the lead author explicitly indicated not to be interested in any kind of moral assessments of the players' stories. Retrospectively, these reflections on positionality were supposed to be crucial for balanced power relations and for data quality. Nevertheless, assessing the extent by which the researcher's “own identity may or may not have interacted with the interviewees' self-perceptions” (Faas, 2009, p. 303) has proved to be difficult.

In order to achieve data saturation, participants were given the opportunity to share further thoughts and reflections at the end of the interviews. When both, the interviewer and participant, felt that there was nothing more to share, we assumed that data saturation was reached with regard to the individuals' stories on their national identity development. However, we acknowledge that our findings might only offer a glimpse into our participants' rich and complex experiences of national identity development, as “to the extent that each life is unique, no data are ever truly saturated: there are always new things to explore” (Wray et al., 2007, p. 1400). Hence, it is important to keep in mind that narrative research is always tentative and that our proposed typology of narratives should not be treated as fixed and exclusive categories (Frank, 2010; Cavallerio et al., 2017; Ronkainen and Ryba, 2019).

### Data Analysis

For data analysis, we used both, the interview data and the biographical mappings. The lead author transcribed all interviews verbatim in German; key statements were translated into English by the authors and checked by a professional translator. The paper-pencil mapping grids for each player were processed and digitalized so that the different line drawings (for the purpose of this study mainly *identification with Germany* and *identification with Turkey*) could be compared during the data analysis.

For data analysis, we adopted the story analyst position (Smith, 2016). The lead author conducted the analysis in regular discussion with the research team, who acted as critical friends (Smith and McGannon, 2018). Data analysis started with a process of “indwelling” during which the lead author read the transcripts and assessed the biographical mappings for each player to identify themes and story plots inductively (Smith and Sparkes, 2009).

We conducted a thematic narrative analysis (Smith, 2016) of the interview data to identify the main themes within each player's story of the national team decision making process and his national identity development. In an initial step, we created case profiles for each player in which we summarized relevant contextual information such as family immigration history, citizenship, identity formations, and (international) junior career stages. Next, the lead author marked the transcripts with conceptual comments, which primarily focused on the semantic and latent content of each story. During this process of categorizing and sorting data, we identified main categories, statements, and themes using MAXQDA software. We specifically focused on the parts of the stories where interviewees made references to their identification with Germany and Turkey before, during, and after having made the decision for which national team they want to play. In a next step, the first author prepared extensive case summaries for each player focusing on his national identity development.

Next, to come to terms with the story's structure, we particularly focused on the biographical mappings, specifically the national identity development lines. For getting to grips with stories, Smith (2016) suggests that researchers depict the story's structure within a graph. Within our study, the biographical mappings served as visual images of the story's structure and were created by the participants themselves. Thus, we supplemented our case summaries with the graphic data from the digitalized mapping grids. During this step, quotes from the interviews that contained specific information as to the strands, turning points, and bifurcations in the national identity development lines (i.e., *identification with Turkey* and *identification with Germany*), were entered into the digitalized mapping grid as well as when the respective player first started to ponder for which national team to play and when he made his decision.

Once these analytical steps were concluded, we aimed to build a typology of stories on national identity development of German-born national youth football players with Turkish background. To this end, in a first step, we systematically compared the national identity development lines within the biographical mappings of each player and the case summaries to identify “the most general storyline that can be recognized underlying the plot and tensions of particular stories” (Frank, 2013, p. 75) with regard to national identity development. To make sure that our developed types of narratives are grounded in the actual participant's stories, we revisited the original interview data.

### Methodological Rigor

As our research is based on an interpretative paradigm, we abstain from employing a “criteriological approach” (Sparkes and Smith, 2009) to assess the quality of our data and analysis. Instead, we perceive quality criteria as characteristics of our research. As a point of departure, we have used the work of Tracy (2010) and Smith and Caddick (2012) and invite researchers to take into consideration the following criteria that align with our study: transparency, sincerity, and worthy topic.

Based on our identity theoretical perspective, we assume that national identity is a particularly sensitive issue for players with migrant background as the players are supposed to be aware of public expectations and media debates on national identity in Germany. Against this background, it is not surprising that McGee and Bairner (2011) resume that “understanding of such matters has been hindered by a marked reluctance on behalf of professional athletes to discuss their experiences with researchers” (p. 439). For this reason, it was assumed to be particularly important to be transparent in the study's aims and to clearly distance from public allegations and normative valuations. Considering that interviewing young people requires specific reflections on ethical issues, we made transparency, confidentiality and balanced power relations our highest priorities. According to the ethical standards of qualitative research, our study was based on the concept of informed consent. Participation in the study was thus entirely voluntary. Confidentiality and anonymity were guaranteed to interviewees and to their clubs. In order to encourage the players to express their views freely and to avoid socially desirable answers, we accentuated the fact that we were not interested in any kind of moral assessments regarding the players' national team choice.

## Results and Discussion

We decided to combine results and discussion. Overall, we identified three different *types of narratives on national identity development* of German-born youth international football players with Turkish background. In the following section, we present one case for each type of narrative to allow for analytical depth and to stay closely to the participants' own stories, while being aware of the fact that a single story does not necessarily contain all characteristics of the respective narrative type (Kuckartz, 2020; John and Thiel, 2022). However, this representational strategy is frequently recommended and employed in narrative studies (e.g., Phoenix and Smith, 2011; Cavallerio et al., 2017). In addition to the interview data, we also refer to the digitalized biographical mapping of the player whose story represents the respective type of narrative. Within the biographical mapping, we have also entered the most important events to which the player referred to when narrating his personal story on national identity development and his football career. Therewith, the biographical mappings serve as visualized reference points that help in understanding the players' stories on their national identity development.

Each type of narrative is characterized by specific features and strands of development. In the following, we characterize and discuss each type of narrative on national identity development by identifying notions of national identity and by interrelating the national identification with Germany (as the country of birth) and Turkey (as the family's country of origin) during the sport career and against the background of the international career. Although the stories are highly individual, we also aim to trace similar features within the players' narratives on national identity development.

### Type 1: “Going With the Nomination(s)”

**Figure 1:**
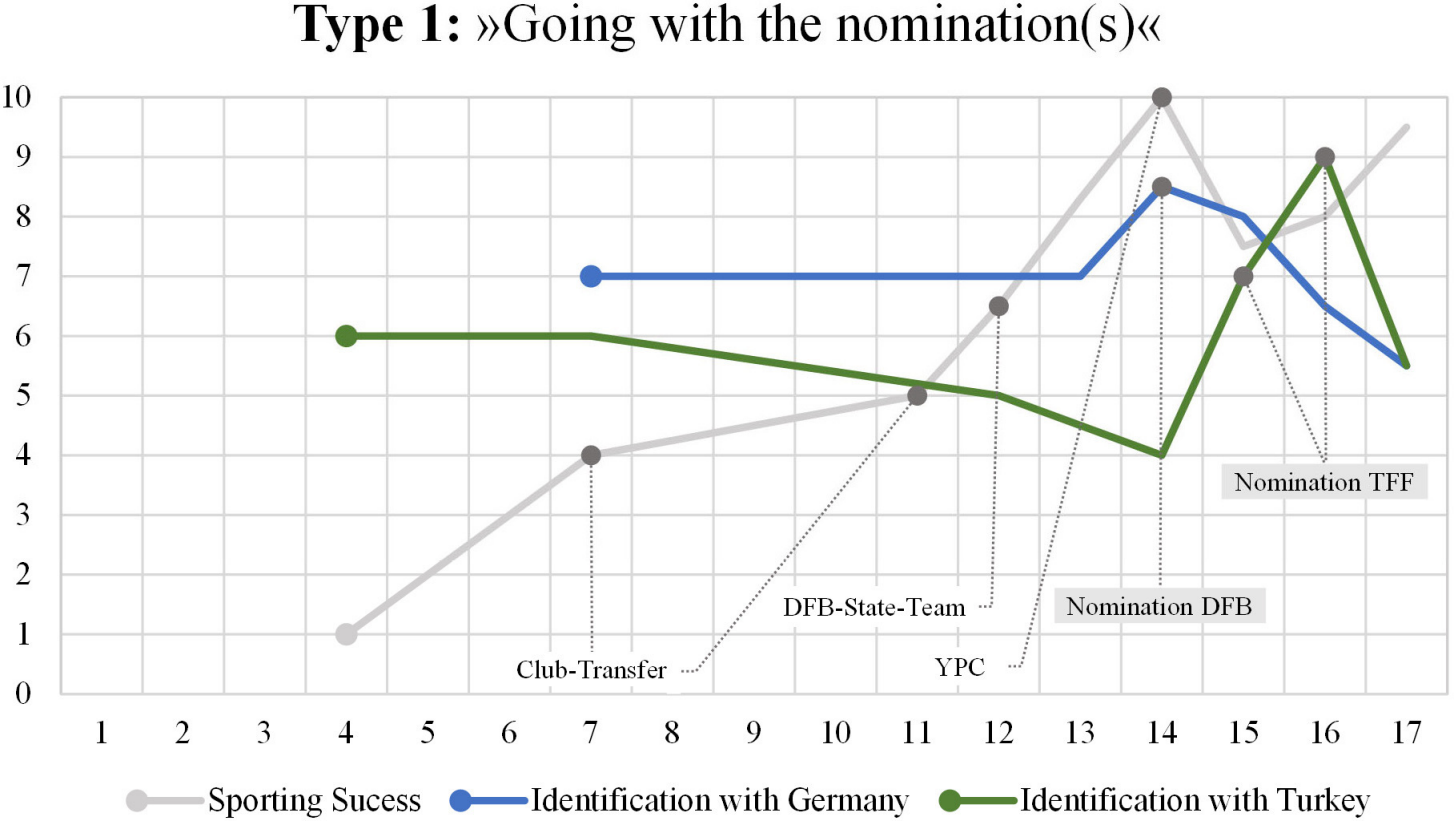
Mapping Type 1.

The “going with the nomination(s)” narrative (Figure 1) is characterized by an inseparable connection between the nomination of a player by a federal football association (DFB or TFF) and the development of national identification with Germany and Turkey. Dynamics in national identity development particularly arise in the context of the national team question. Typically, national identification is attached to being recruited for a national team; here, the identification with the nation that recruited the player for an international game or tournament rises significantly. At the same time, this rise is accompanied by a decline of national identification with the other nation. The following biographical mapping of Emir (name anonymized) is typical for that type of narrative.

Emir is a descendent of a former “guestworker”. His grandfather immigrated to Germany in the course of the big labor agreements with Turkey at the end of the 1960s. Just like his grandfather, his parents were born in Turkey and immigrated to Germany during childhood in the early 1970s. The player is the first generation of his family who is born in Germany. His first language is German, his second language (albeit at a lower level) is Turkish. Although he reports to have a dual citizenship, nationality is not a relevant personal issue for him. Nevertheless, as the German Football Association (in contrast to the TFF) defines German citizenship as a recruitment condition on the level of national teams, his nationality status qualifies him for playing for both national football associations. Actually, during his junior elite sport career, the player was recruited for several national junior teams by both national football associations (DFB and TFF). Generally, Emir describes himself as a “German-Turk”, but at the same time he points out to rather have a strong local identity that is closely connected to his city of birth in Germany. His bi-national references are illustrated by sport related examples—such as supporting Germany and Turkey during World Championships and by placing both national flags at the car of their family during the tournament:

“*Even at the World Cup and so on, we have, for example, Germany and Turkey, these flags on the cars, we had Germany and Turkey. So (.) there is not such a difference.”*

Looking at the strands of national identification in his biographical mapping, it is evident that the reconstruction of national identity starts in (early) childhood. Obviously, the player's story of national identification with Turkey starts earlier and on a lower level than the story of national identification with Germany. When asked about this issue, Emir marked this as an unconscious differentiation. His appointment by the German and later by the Turkish football association were reported to be massive boosters for his national identity development. Both peaks in his biographical mapping result from being nominated for international games or tournaments. The first international game for Germany represented a crucial point in his national identity development story. The rise and peak of the national identification with Germany was explained by his surprise and satisfaction of being selected by the DFB for a game with the junior national team. Emir recounted:

“*With Germany, it was like, maybe I was so happy because I never thought I would play for the junior national team.”*

He went on and specifically compared the feelings he had when being nominated for Germany with the ones he experienced when being invited to play for Turkey:

“*Yes, when I (…) have sometimes seen who was invited to Turkey, so (…) I was now not so much pleased; so also already very pleased, nevertheless for the invitation [by the TFF], but not so much as for Germany, because actually I was there still a player of [name of a small football club]. This was also still on the list for the DFB training course. There were always the players, there stood Borussia Mönchengladbach, VfB Stuttgart, Bayern Munich and then there was I from for example [club name]. Among the Turks who were from Germany, there were many from smaller clubs who were invited. Yes. So, at first I thought it was better [to play] for Germany.”*

A special feature of this type of narrative is that the story of national identity development is inextricably linked with the interest of and the act of being recruited by a national football association. Changes in this development process result from either being nominated or not being nominated. Being nominated leads to a rise of identification with a national football association and a nation. Conversely, if a national football organization loses interest in a player and does not nominate him for a current training course or international game, the level of national belonging rapidly sinks to a level that is even below the initial level. In the interview, Emir explained the decline in his national identification with Germany with his disappointment about not being recruited for the next international game of the DFB and the feeling of not being treated fairly by the coach of the German youth national team.

“*Yes, until [a certain game] I was in the A squad. [...] Yes, and then there was an international match against [nation] and then my competitor on my position was invited, even though he was not in the training camp for two weeks. That's why I didn't want to play anymore. (...) That's why I was angry, because he wasn't there for two weeks and in [nation state] he was, when we had two international matches. That was so obvious. (…). So after each game we were graded and in [nation state] he once gave me the same grade as my competitor even though he played much worse and many have seen that. That they did unfair things.”*

Against this background, it seems appropriate to point out that we find similar episodes in various interviews. Accordingly, another athlete who played for the TFF first and then for the DFB reports:

“*But as I said at the beginning, the Turkish national team (-) the Turkish players from Turkey were preferred and I perceived that as a disadvantage and not so positive. And that's why I also directly (-) so when I came back, I also said to my parents that I want to play for the German national team. And since then, I haven't played for the Turkish national team.”*

It can be concluded that national identity development coalesces with athlete identity and the performance logic of elite sport (Brewer et al., 1993; Carless and Douglas, 2012). In line with our theoretical assumptions, Emir's story shows that national identity development is framed and triggered by the social context of youth elite football. Accordingly, changes in the strands of national identification are presented from the angle of a youth elite athlete striving for an international career and, for that reason, conceptualizing nominations for international matches as major events in terms of national identity development.

A further characteristic of the “going with the nomination(s)” narrative is that national identity is not a stable construct as can be seen within the player's biographical mapping. When referring to one specific experience he had after an international junior game with the TFF, Emir offered the following account:

“*Last year, after the European Championship qualifiers [TFF], we were at the airport and our Turkish national team coaches, I don't know from where, also heard that two or three players had been invited [by the DFB]. Yes, the coach said that, but I also thought he was right, 'whoever goes there won't be invited to the Turkish national team, because you all think we're only second choice and we don't need players like that', he said. I thought he was right. Then I think two players left anyway. They weren't invited back for a while, but they were invited back the other day.”*

Although, the player agreed with the national coach who tried to establish the emotional identification with Turkey and the TFF as an unconditional commitment, this experience, however, did not lead to a stable national identification with Turkey afterwards for Emir. As the player was not nominated by the coach and the TFF, his identification with Turkey sinks significantly again.

“*In Turkey [at the TFF] I wanted to stay for the time being, but I don't know (-) Now there was a tournament the other day and I just wasn't invited.”*

The second decline of the national identification with Turkey line indicates that the emotional attachment to Turkey as a nation seems to be binding just as long as the TFF decides to recruit the player for the next international games of the age group.

Against the background of this story, national identification development in German youth elite football appeared to be fundamentally shaped by elite sport socialization and by transnational talent recruitment in elite football. Besides, the story is characterized by variability, volatility, and instability. It is the current status of being and of being not recruited for international games that initiates short-term changes in the identification with Turkey or Germany. Within this type of narrative, national identification is conceptualized as a highly variable and situative construction that is, in line with previous research, crucially affected by “sporting pragmatism” (McGee and Bairner, 2011, p. 442). This type of narrative is an excellent example for the fluidity of national identity in the context of elite sport. Apparently, this fluidity results from processing the recruitment decisions of the national football associations and the experiences during the national training courses. Within the “going with the nomination(s)” narrative, national identity development can be compared with an elevator that drives up and down and that is steered externally by athletic events or non-events such as nomination and deselection for a national team. Consequently, national identities are under threat if former nominations are not refreshed. Accordingly, the quality of identification with Germany and Turkey is primarily driven by recruitment decisions of the TFF or the DFB. In this sense, national identity is functionally linked with the prospect of success and of perceptibility in elite sport. Accordingly, the identification with a nation rises when there are constellations or options to increase the players visibility.

“*So, you can already see when you're playing for Germany right now (-) you're already seen more because they're more often at the World Cup and so on. European Championship, World Cup and so. And at the moment they are also more successful.”*

### Type 2: “Reconsidering National Belonging”

**Figure 2:**
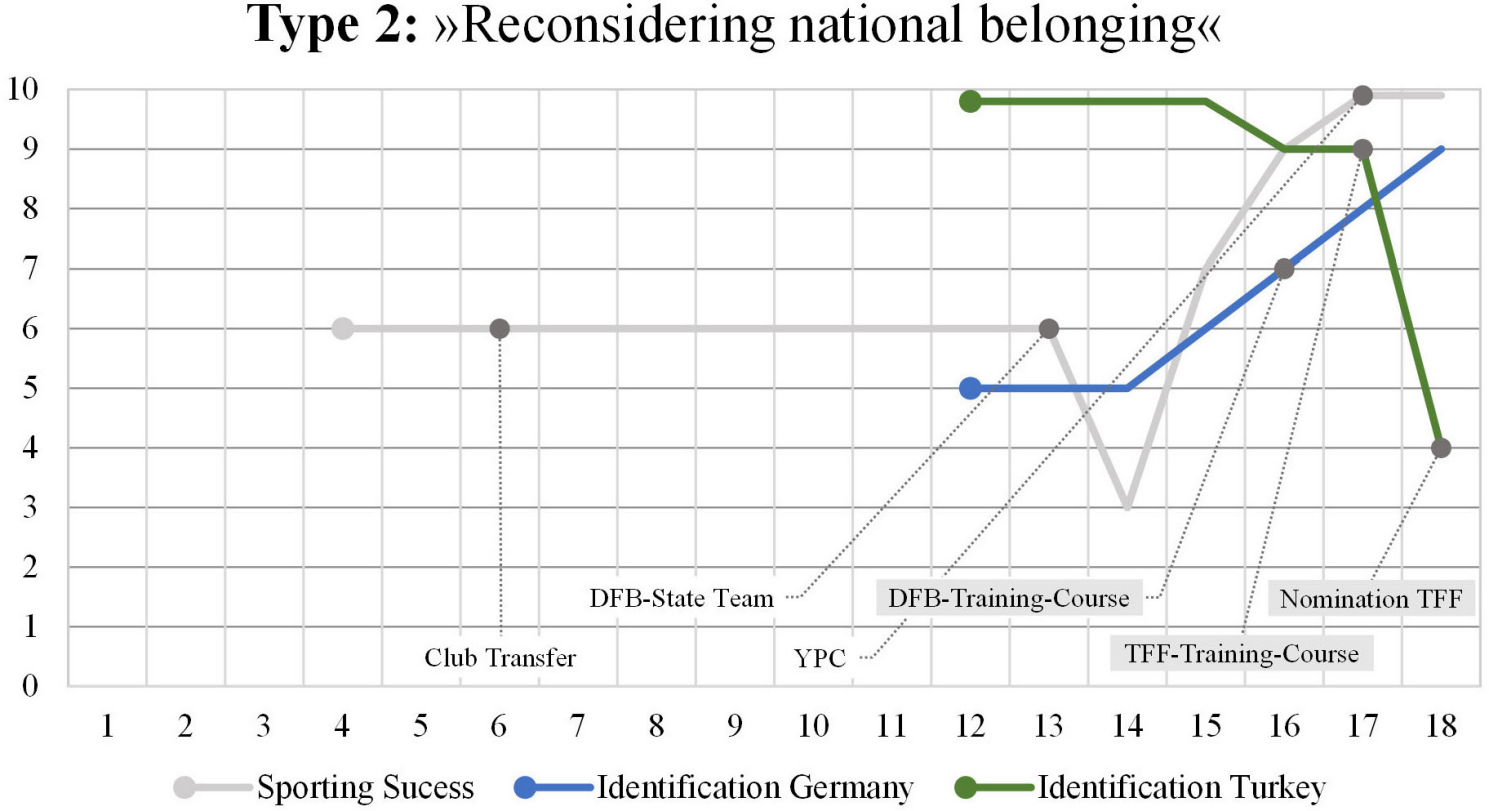
Mapping Type 2.

While the first type is characterized by the rise of the national identification with the nation which had nominated the player for international games, the “reconsidering national belonging” narrative (Figure 2) is characterized by a reverse dynamic. In the aftermath of being recruited for a specific nation, the identification with this nation declines rather than rises as it is the case within the “going with the nomination(s)” narrative. This reverse dynamic typical of the “reconsidering national belonging” narrative can be seen within the biographical mapping of Cem.

Within his family's immigration history, Cem belongs to the third generation. It was his grandmother who immigrated from Turkey to Germany as a working migrant at the beginning of the 1970s and reunited the family later in Germany. The player's parents were born in Turkey and immigrated to Germany in childhood. Thus, the player is the first generation being born in Germany. He has a Turkish passport but not the German citizenship. At the time of the interview, Cem tells about having applied for German citizenship and planning to return his Turkish passport then. Although Turkish is the dominant family language, the player speaks German und Turkish fluently. With regard to his football career, Cem was intensively involved into the federal talent development program of the DFB. He trained at a local DFB-base, later at a YPC and played for German junior state teams. On a junior international level, he initially was invited for two national training courses of the DFB before he chose to play for Turkey. Subsequently, he played for the TFF in several junior teams.

Cem's graphical reconstruction of national identity development starts in late childhood. It is evident, that the two lines start at the same time but on absolutely different levels of national identification. While the identification with Germany is marked on an intermediate level, the identification with Turkey is mapped on a maximum level. In his verbalized story, his national affiliations with Turkey became apparent when he described himself as a “Turk”.

“*If I'm in Germany, or people in the street ask me, ‘What are you? German or Turk?', of course I answer I'm Turkish.”*

A recurring theme in his story of national identity development is “Heimat” (a German word that cannot be directly translated to English, but approximately describes a sense of emotional belonging). This term is very present when speaking about Turkey as his family's country of origin. For example, when speaking about his parent's city of birth in Turkey, he uses the term “hometown”.

It is evident that this term has an emotional connotation for the player. It can be assumed that this emotional attachment with the “home country” Turkey is the reason why his visualized story is characterized by a very strong affiliation with Turkey. It can be assumed that this emotional identification with Turkey was transferred to elite sport by the idea of someday playing for Turkey. Cem recounted:

“*My dream was always to play for Turkey. That was just this decision, because I wanted to fulfill my dream that I had as a child.”*

“*My mother really wanted me to play for Turkey, and my mother is everything to me, and that was actually the thing where I said ‘yes' to Turkey.”*

While both national identification lines are stable in late childhood, the interrelation between the two lines starts to change at the beginning of adolescence. While the affiliation with Germany rises, the sense of belonging to Turkey significantly falls. The rise of the identification with Germany fell into a career period in which Cem became Junior-Bundesliga-Player, changed the club, and started his international junior career for the TFF. The two lines cross at the time when the player transferred to his new club and its YPC.

This major switch is particularly explained by experiences the player had during his international junior career with the TFF. In contrast to Type 1, the nomination for the TFF did not result in an intensification or assurance of national affiliations with Turkey but initiated a process of revision and regression. Crucial for Cem's story are some experiences he made during the training courses and international games in Turkey. He experienced the training courses and the selection of starting-players to be less performance-related, disciplined, and transparent than he was used to at his German club.

“*So (.) for example (.) the Turks rather do not look at the performance of the other players, there have played very many who have not earned it or no idea, it was just very strange, not (-) so to speak not as much discipline as the Germans and I just did not like it and I'm just used to the Germans with this discipline that only those play who also perform.”*

Besides, the story also revealed experiences that seemed to irritate his emotional identification with Turkey and his self-description as a “Turk”. Apparently, his trips with the national team and the trips to Turkey intensified his feeling of not being perceived as a Turk in Turkey. This feeling of unfamiliarity and not belonging is reported to be a major turning point that made him rethink/reassess his understanding of “Heimat”, his emotional commitment to Turkey and his general affiliations toward the two nations:

*If I'm in Germany, or people in the street ask me, ‘What are you? German or Turk?', of course I answer I'm Turkish. However, if I'm in Turkey, people there say, ‘You're not a Turk.' Most people say, ‘You're a German Turk. You're German.' They don't see me as a Turk. Even if I go to my hometown [in Turkey] they tell me this.”*

Apparently, processing these experiences led the player to recheck his affiliations with Turkey and to rethink his emotional linkage. This finding is in line and, at the same time, not in line with current research on national identity assuming that “[w]hen individuals experience a sense of belonging by means of self-categorization as group members and then become aware that their fellow members share the same identification, their world changes” (David and Bar-Tal, 2009, p. 371). On the one hand, the player categorized himself as a “Turk” and defined himself as a member of a national group. On the other hand, he experienced to be not perceived as a Turk by this group. As a consequence, his “world changes” indeed—but not in the way that his national identification with Turkey rises. Instead, processing these experiences led to the recognition that Germany actually provided more reference points for national identity than Turkey:

“*I'm in Turkey just, let's say, every other year; thus, I'm not there often. I don't really see Turkey as my home country since I'm never really welcome. In Germany, I feel comfortable. Actually, I see Germany as my home country. Sometimes I even ask myself, why I say ‘I'm Turkish' although Germany offers me everything I want and I feel comfortable here.”*

### Type 3: “Adding Up Chances”

**Figure 3:**
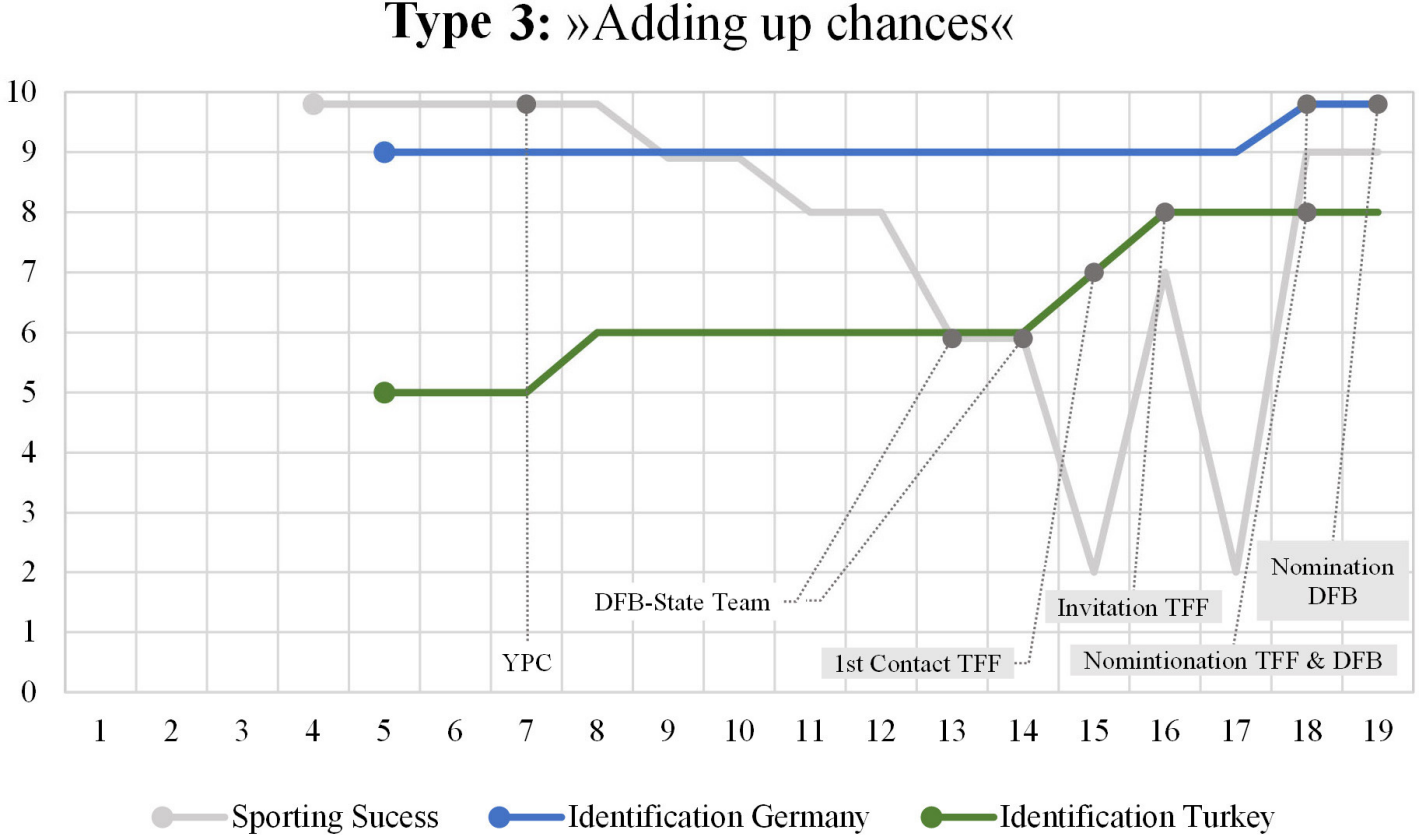
Mapping Type 3.

The “adding up chances” narrative (Figure 3) is characterized by an increase of both strands of national identification, that with Germany and that with Turkey. In contrast to the first two types of narratives, these stories do not include (significant) decreasing trends with regard to national identification with one nation. The participants whose stories follow the “adding up chances” narrative particularly refer to the chances and opportunities in their (sport) biography and highlight the resulting positive effects on their national identity development. Although the level of identification with Turkey and Germany differs to some extent, national identity development its the result of adding up these chances and effects. The “adding up chances” narrative can be seen within the biographical mapping of Murat.

The player's migrant background results from his father's family who originally comes from Turkey. His fathers' grandparents immigrated from Turkey to Germany—and went back to Turkey later on. His father was born in Germany. Murat's mother is German without having a migrant background. The German-born player is German citizen, which gives him the option to play for both national football federations. He does not have a Turkish passport. His first language is German. He barely speaks Turkish. On a level of self-positioning, he describes himself as rather German than Turkish. In terms of his football career, Murat played for a YPC and for a state team of the DFB. In the course of his international junior career, he was (at different times) nominated for Turkey (TFF) and for Germany (DFB).

The visualized story of national identity development starts in early childhood. Looking at the two strands, it is evident that the level of identification with Germany is constantly marked higher than with Turkey. At the same time, Murat reports to also have a strong local identity that refers to his city of birth in Germany. Generally, Murat's story implies several references to his “subjective sense of togetherness, we-ness, or belongingness” (Turner, 2010, p. 16) to Germany and to Turkey:

“*Well, I would see myself a bit more as German, because I don't know the language either, the Turkish language. But so, so I have a bit of Turkish blood in me, so in the emotions sometimes, but yes, of course I see myself more as a person from [name of a German city] than a Turk, quite clear. [...] Well, I see myself as German, but it wouldn't also be difficult to identify myself as Turkish. This wouldn't be a problem. I get along with the [Turkish] people. They are kind-hearted. Therefore, I see no problem there.”*

Within Murat's story, Turkey represents the residence of the relatives on his father's side. Since his childhood and despite the hyperinclusion into elite youth football the player travelled to Turkey almost every year for vacation in order to meet and spend time with his grandparents and his father's family of origin.

“*From the private side, it was always so that we went to Turkey almost every year for vacation, to the relatives. Whether it was now the uncle of my dad or just my grandparents, no matter whether it was now in [name of a Turkish city] directly, I have actually always felt very connected with Turkey.”*

Several players reported a strong attachment with Turkey resulting from family holidays in Turkey. The following statement of a Turkish international junior player, however, points out that this emotional attachment decreased as the player had to invest more time resources into youth elite football. This biographical fixation stopped him from traveling to Turkey and to sustain his emotional attachment:

“*The connection with the homeland [Turkey] started relatively early because (-) we were often in Turkey when we were young or I was often in Turkey when I was young. (...) Yes, then it [the connection to Turkey] was actually relatively lost. Through soccer, because I personally was no longer so often in Turkey.”*

Within the case of Murat, the rise of the identification with Turkey line is explained by two aspects. Firstly, the player reported to be impressed by the TFF's commitment. Since 2010, the TFF was in contact with the player and his parents. In this context, the player tells about meetings with the head of the European Office (TFF) aiming to convince the player and his parents of playing for Turkey. Apparently, the first invitation for a national training course of the TFF was refused by the player's club. A second reason for an increased identification with Turkey is that being invited and nominated for international games by the TFF amplified Murat's feelings of belonging to Turkey. The nomination and the experiences around the international game initiated a process which intensified and emotionally charged his relations with Turkey sustainably.

“*Of course, it [the identification with Turkey] became stronger when I was invited here, then I listened to the anthem so that I knew the melody at least a little bit [laughs] and then my interest for Turkey clearly became stronger, because I somehow also wanted to be interested in it, wanted to know a little bit more about the country than I knew before, but (-) and that actually continues until now, that I now also root for them or root more for them now when they have a game, that I then also get upset why they do not win now. That they are not in the World Cup, for example, that makes me very sad. I would also quite clearly cheer for them, no question. So in general, the interest in Turkey or the attachment was actually relatively high because we were there actually every year.”*

The identification with Germany line already starts at a high level and is linked with the feeling of being rather “German”. From early childhood until late adolescence, this line is very stable. Interestingly, and in contrast to the other two types of narratives on national identity development, being invited by the TFF (and not by the DFB) in the first place, did not have an impact on Murat's identification with Germany at all. The peak of the identification line coincides with the nomination by the DFB. To wear the official dress and the feeling of representing his country of birth are told to be boosters of national identity development.

“*Yes, that's actually from the beginning, I would say maybe at nine [the identification with Germany] and then where it got straight down to the nitty-gritty, with the national team, that I have then properly identified with it, and that I then just run around with the clothes that I represent my country (…). That was actually the greatest for me, is still the greatest for me.”*

Another athlete who played exclusively for Turkey tells quite a similar story:

“*So I was relatively proud of myself that I got an invitation from the national team, the Turkish team. Yes, I remember exactly how much I was looking forward to it. And when I played my first game, when I was already wearing the jersey, I still remember how it made me tingle. Of course, when I listened to the national anthem, for me, that was such a goosebump feeling. That's when I realized how connected I am to my homeland [Turkey].”*

Generally, within the “adding up chances” narrative, the identification with Germany and Turkey lines are comparatively stable. The biggest leap in Murat's story resulted from the nomination for the TFF. Characteristically, not being nominated initially by the DFB and later by the TFF did not affect his feelings of belonging to a nation. At the time of the interview, the initial difference between the two identification lines is significantly reduced. Typically, within the “adding up chances” narrative, having reached a certain level of national identification means that this level is at least maintained. National identity is conceptualized as a rather stable construction that is reinforced by becoming an international player. In this type of narrative, youth elite football offers various chances for processing national identification. Interestingly, national identity is presented as a growing resource that is fed by the opportunities that go along with a migrant background and an international career in youth football. In the course of this process, experiences are positively aggregated, which is typical for hybrid identity formations in immigration countries (Faas, 2009).

## Conclusions

In contrast to previous analyses that asked for the role of national identity and network actors for the national team question of German-born international youth football players with a Turkish background (Seiberth's et al., 2017; Seiberth et al., 2018; Seiberth and Thiel, 2021), the aim of this analysis was to explore the national identity development stories of these players. By conducting and analyzing 10 expert interviews and biographical mappings, we constructed a typology of narratives, namely “going with the nomination(s),” “reconsidering national belonging,” and “adding up chances”, that players rely on when constructing their personal stories.

Generally, the study confirms the assumption that national identity “is not a static foundation passed down from generation to generation” (David and Bar-Tal, 2009, p. 373). Rather, our findings illustrate that national identity development in youth elite sport is a highly individual and dynamic process that is particularly shaped by the experiences in the context of a career in youth elite sport. In this sense, the stories describe (German) youth elite football as a specific social context that “provides multilayer conditions of different types, scopes, qualities in which individuals and collectives operate” (David and Bar-Tal, 2009, p. 371).

In line with current research, the stories on national identity development mostly start in (early) childhood. Regarding this early stage of (national) identity development, the stories include episodes of relevant family-related experiences, like family holidays in Turkey, or of the family supporting a specific Turkish football club. Although it can be assumed that these experiences had an impact on personal national identity formations, this pre-elite-sport period is characterized by a rather constant level of national identification with Turkey and Germany. Apart from that, our findings indicate that the specific family background or the status of the parents (e.g., being born or not being born in Germany) did not play a specific role for the players' national identity formation. In fact, the stories rather show that several of the interviewed players only have very limited knowledge about the family's immigration history. Furthermore, the increasing commitment to elite sport usually leads to a decreasing contact frequency with the family. At the same time, the contact frequency with elite sport actors (such as coaches) rises (Seiberth and Thiel, 2021).

Generally, the stories indicate that significant changes in national identity development take place in adolescence. Indeed, the closer the players come to the age when the national football associations start to recruit their players, the higher are the dynamics within the strands of national identity development. On the one hand, this can be explained by an increasing hyperinclusion into elite sports that leads the players to focus on the next career step “junior national team” and for that reason to closely attach constructions of national identity to the national team question. On the other hand, is must be considered that dealing with aspects like ethnic origin and reflecting on one's relation to the country of birth and the country of one's ancestors is typical in adolescence. However, whether the dynamics of national identity development are age-specific consequences of processing questions of ethnic origin and national belonging or an effect of the recruitment structures in junior elite football (starting with the Under 15-Team) is hard to tell. We assume that both effects aggregate if a player is nominated by one or both national football associations.

In fact, our study shows that erratic changes of national identification are always coupled with becoming an international player, but that the nomination for a nation does not reliably and sustainably booster feelings of belonging to that nation. Hence, the stories indicate that international careers not necessarily foster national identification with a nation but can also reduce feelings of belonging to a nation—even with the nation the athlete plays for. Particularly negative experiences with the national football federation or in the context of international games have the potential to reduce national identification with that nation.

This brings us back to a basic assumptions of identity theory and biographical research, pointing out that individuals process such experiences and life events differently and come to alternative conclusions (John et al., 2019). The three different types of narratives within our study illustrate this impressively. Overall, the study confirms that sport is indeed “an identity forming factor” (Dóczi, 2011, p. 3)—one way or the other. Nonetheless, since we assumed public and media narratives to play a role within the players' stories and although we mentioned these media narratives during the interviews, it was particularly surprising that such master narratives were not marked as “identity forming factors” in the stories. We assume this finding to be closely connected to the dominant role of athlete identity in youth elite sport (Schubring and Thiel, 2014; Seiberth's et al., 2017). Apparently, this elite sport identity not just shapes the national team question sustainably, but also has the potential to override other narratives. In this sense, athletic identity becomes a driving force for processing national identity formations in youth elite sport. While non-athletic adolescents with migrant background tend to deal with issues of national identity incidentally, youth athletes with migrant background are forced to deal with this issue (at least at the career level) as soon as the national team question arises. We conclude that, although the concept of nation states has dwindled in importance the study highlights that nation states in elite sport still are “powerful identifiers” (Brubaker and Cooper, 2000, p. 16).

Finally, the study needs to be discussed in the light of its limitations. One limitation refers to the number of participants. Apparently, our sample consisted of a comparatively small number of athletes in German youth elite football. The number of 10 interviews resulted from our explorative design that aimed to reconstruct individual stories of national identity development in youth elite sport and to identify specific types of narratives. Rather than focusing on representativity, we aimed to explore the process of national identity development in detail. Accordingly, the ten athletes are not a “representative sample, but rather a contrastive selection of youth elite athletes” (Schubring and Thiel, 2014, p. 81). However, it has to be considered that the interviewed players belong to the group of absolute top performers in German youth elite football. This group is not just hard to recruit but also limited in its number.

The second limitation refers to the fact that the study focused on youth players with Turkish background only. We decided to address this specific population because people with Turkish background represent the biggest ethnic minority group in Germany and are highly represented in German youth elite football (Seiberth's et al., 2017). Nevertheless, focusing on this specific group of youth elite football players further reduced the number of potential interviewees significantly. Knowing that the number of German-born international youth players with Turkish background training in YPCs in Germany is very limited and having identified only 31 players overall who matched the selection criteria at the time the study was conducted, 10 interviews and mappings are supposed to be a solid basis for a first exploratory analysis. Nevertheless, we recommend future studies to include athletes with different migrant backgrounds.

A strength of our narrative approach was the triangulation of methods and data by combining expert interviews and biographical mappings. Therewith, we have extended current narrative approaches that tend to focus only on the verbal story through incorporating a graphic depiction of developmental processes created by the participants themselves (i.e., the biographical mappings). The retrospective on their career and on national identity development in particular gave the participants the opportunity to tell their story with a temporal distance and to analyze the development of national identity over time. Such a processual and narrative perspective adds to current research on national identity formations in (youth) elite sport (Grainger, 2006; McGee and Bairner, 2011; Seiberth et al., 2018). However, in line with our narrative perspective, stories on national identity development represent contingent constructions of reality that likely evolve in the light of future events. Hence, types of narratives are always tentative (Frank, 2010; Ronkainen and Ryba, 2019). Future studies could aim to identify and characterize other subtypes of narratives to expand our understanding of the dynamics, complexity, and ambiguity of “identity work” (Andersson, 2002) in (youth) elite sport even further.

## Data Availability Statement

The original contributions presented in the study are included in the article/supplementary material, further inquiries can be directed to the corresponding author.

## Ethics Statement

Ethical approval was not required for this study.

## Author Contributions

KS and AT contributed to conception and design of the study. KS organized the data collection and the database and wrote the first draft of the manuscript. KS, AT, and JJ performed the data analysis. JJ and AT wrote sections of the manuscript. All authors contributed to manuscript revision, read, and approved the submitted version.

## Conflict of Interest

The authors declare that the research was conducted in the absence of any commercial or financial relationships that could be construed as a potential conflict of interest.

## Publisher's Note

All claims expressed in this article are solely those of the authors and do not necessarily represent those of their affiliated organizations, or those of the publisher, the editors and the reviewers. Any product that may be evaluated in this article, or claim that may be made by its manufacturer, is not guaranteed or endorsed by the publisher.

## Footnotes

^1^According to the German Federal Statistical Office more than 26% of the German population currently has a migrant background (Statistisches Bundesamt, 2022). Although the distinction “migrant background” is frequently used in German administration, politics and migration research there is a critical debate in social sciences that relativizes the explanatory value of the term (Thiel and Seiberth, 2017). Having a migrant background means that a person “or at least one parent did not acquire German citizenship by birth” (Statistisches Bundesamt, 2022). Among the population with migrant background, people with Turkish background represent the biggest ethnic community in Germany. At the same time, and curiously, it also represents a “particularly under-researched community” (Faas, 2009, p. 303).

^2^Evidently, these debates are associated with the political and social trend of growing nationalism, national isolationism and particularism. Particularly, the constitutional referendum of the Turkish president Erdogan in 2017 reinforced a controversial debate about (dual) citizenship, sense of belonging and national identification of people with Turkish background in the German public. This debate was re-launched in 2018 when some newspapers published photos showing the German international football player Mesut Özil familiarly signing a club jersey for the Turkish President Erdogan just before the FIFA-World Cup 2018 in Russia. These photos had significant consequences as they intensified the public debate on national identification of people with Turkish background and as they made Özil break with the DFB.

^3^The media debate on Mesut Özil is an excellent example for this. Although, Mesut Özil opted to play for Germany during his career and despite the fact that he justified his decision several times by referring to his cognitive and emotional identification with Germany as his country of birth and his home country, he got in the center of a media debate criticizing some German internationals with migrant background for not singing the national anthem before the start of international games (Seiberth's et al., 2017; Seiberth et al., 2018).

^4^This methodological decision resulted from the fact that neither the status “migrant background” nor the fact of being born in Germany allows to draw conclusions on the level of citizenship. In fact, being born in Germany does not automatically lead to German citizenship. In 2000, German citizenship law supplemented the principle of descent the principle of birthplace (Gerdes et al., 2016). Since then people with migrant background had the choice to decide whether they want to acquire the German citizenship or not. Until today, among the highly heterogeneous group of people with migrant background there is a comparatively high number of people who are born and socialized in Germany without having a German or dual citizenship (Statistisches Bundesamt, 2022).

^5^Since 2004, FIFA allows players to switch between junior national teams and national football associations up to the age of 21 until they play for the senior national team in a competition match. This is particularly important for players with migrant background as the FIFA statutes entitles them (under specific conditions) to represent more than one national football association. One recruitment condition is that a player's “biological mother or biological father was born on the territory of the relevant association” (FIFA, 2016, p. 70). However, previous research indicates that the DFB's and TFF's recruitment strategies differ substantially when it comes to citizenship. While the DFB is reported to exclusively recruit players with German citizenship, having a Turkish citizenship is not required by the TTF (Seiberth's et al., 2017).
